# A Review of the Present Position

**Published:** 1920-10-09

**Authors:** 


### PREVENTION OF PUERPERAL SEPSIS.

### A Review of the Present Position.

A STUDY of the statistical tables of the Registrar-General dealing with the question of puerperal infection reveals the fact that the science and art of obstetrics does not in this respect reach the high-water mark of perfection at which it aims, and that even to-day the risks of sepsis to the parturient woman are very real. At the recent annual meeting of the British Medical Association, the proceedings of the obstetric section were chiefly devoted to the discussion of this problem, and some exceedingly important papers were read and discussed.

### THE ORGANISMS RESPONSIBLE.

In nearly all severe cases of puerperal sepsis the streptococcus, either alone or in conjunction with bacillus coli communis, is the predominant organism. The infection may be autogenous or heterogenous—that is, the organisms may have been resident in the patient before the confinement, or may have been introduced from without during its course or afterwards. Which of these happenings is the commoner is rather an unsettled question, but Mr. Victor Bonney holds that intrinsic infection is the great cause of the present-day puerperal sepsis, a theory which opens up a very large field for investigation and research.

Infection from extrinsic sources was no doubt the cause of the epidemics of puerperal sepsis which in the past periodically decimated lying-in hospitals; these the introduction of antiseptic midwifery has succeeded in abolishing, but at the present day it is certainly still operative in those instances where a series of cases of puerperal fever occurs in the practice of a midwife or doctor. But how are we to explain the sporadic occurrence of the disease at the present day in cases where every possible care is taken to avoid extrinsic infection? Antiseptic measures are almost universally used in obstetric work at the present day, and have been for many years; yet when we examine the result we find to our surprise that although the epidemics of puerperal sepsis that were common in the days before antisepsis scarcely occur now, yet the yearly mortality due to the disease, not only in this country but in all countries, shows a very unsatisfactory degree of diminution as compared with the results obtained by antisepsis in surgery.

The obvious deduction to be drawn from these facts seems to be that the antiseptic precautions in use up to the present time have been efficient in preventing that mode of infection in which organisms are conveyed from one patient to another, but that there is some other mode, far more common, for which they are inadequate.

### DO ORGANISMS PRE-EXIST IN THE PATIENT?

Do organisms capable of producing puerperal sepsis commonly pre-exist in the woman? Mr. Victor Bonney says the answer is Yes, and declares they can be constantly isolated from the lower bowel and peri-anal skin. The extensive study of infected gun-shot wounds during the recent war showed that the more virulent bacteria obtainable therefrom were, in general, excremental in origin—that is, they were derived either from the individual's own fæces or from the fæces of some other individual, or from the fæces of some animal in the form of manure.

In May 1919, when Mr. Victor Bonney first put forward the views given above, he encountered very strong opposition. To quote his words, "The principal arguments of those who defended the outlet of the intestinal canal from the aspersions I had cast upon it appeared to be: first, that through ages of evolution it had always been next door to the vagina and at times coterminous with it, and therefore, being natural, must be harmless; and, secondly, that fæcal contamination of perineal lacerations frequently occurs, and healing by first intention takes place as a rule. To the first argument, which reminds me of the philosophy of Crichton in Barrie's classical play, I would reply that Nature makes for death no less surely than she makes for life; but the second requires closer discussion. It is true that tissues accustomed to fæcal contamination show a strong resistance to infection by intestinal organisms; if it was not so, successful intestinal anastomosis, for instance, would be impossible, and all operations on the anus would be bound to end in necrosis or suppuration. But the case is very different when intestinal organisms are implanted in tissues to which their presence is entirely foreign. This knowledge, which has been gradually crystallizing out of surgical experience, obstetricians would have come by sooner had midwifery been taught and practised as the branch of surgery which it is."

### HOW DO THE ORGANISMS GET INTO THE UTERUS.

In this regard, attention should be drawn to the remarkable address delivered by Mr. C. J. Bond at the meeting of the British Medical Association at Leicester as far back as 1905.

He detailed certain experiments carried out by him, which showed that minute particles of indigo, carmine, or litmus placed in the cervix and upper vagina could be detected in the Fallopian tubes in about twenty hours afterwards, having been apparently carried up by an ascending current in the secretion from the wall of the uterus. There would appear to be a current along the surfaces of these canals flowing in the reverse direction to the main stream of the contents, analogous to that along the bank of a swift-flowing river. Bond's work would seem to offer a complete explanation of the fact that the symptoms in a large proportion of the cases of puerperal sepsis appear much later than they should do if the disease were due to direct implantation of organisms on the uterine wall during labour.

### PROPHYLAXIS AGAINST SEPSIS.

The principle of antiseptic surgery as conceived by Lister was the creation of antiseptic conditions,

**Prevention and Treatment of Puerperal Sepsis**—(*cont.*).

in the wound primarily, and, therefore, as a corollary in all that surrounded and touched the wound. To prevent the conveyance of organisms from the adjacent skin into the wound the up-to-date surgeon attaches towels or sheeting in such a way as to cut the skin out of the operation area altogether. It is impossible fully to apply this method to the vagina in labour, but at least the anus should be excluded in all operative midwifery by fixing over it, either by clips or stitches, a large gauze pad soaked in a strong non-irritant antiseptic.

"Violet-Green."

Professor C. Browning and Mr. Bonney have shown that practical sterilisation of the ano-perineal skin can be effected by the use of "violet-green"—a 1 per cent. solution of equal parts of crystal violet and brilliant green in half-and-half alcohol and water—and it has recently been suggested that this antiseptic should be used to paint the vagina and its approaches prior to any operative procedure, and that it should be used as the lubricant every time a vaginal examination is made. Mr. Eardley Holland has suggested that vaginal examinations be done away with altogether and rectal examination substituted in its stead; this mode of examination having all the advantages of the older method and none of its disadvantages. The recto-vaginal septum is thin and lax, and the state of the cervix, the sutures and fontanelles, the degree of descent of the head in the pelvis, and even a prolapsed cord, can all be felt with the utmost clearness. Furthermore, rectal examinations are infinitely less trouble, for none of the aseptic ritual, so necessary in vaginal examination, is required, and the number of examinations need not be restricted.

Intra-uterine Douching.

One of the recognised treatments for many years has been the douching out of the uterus and the removal of "retained products" from its cavity by means of a blunt curette. This practice, however, has been recently condemned by some observers, on the ground that the antiseptic used is so weak that when mixed with the uterine discharge it is doubtful if it could have any lethal effect on organisms merely in the uterine cavity, and certainly none on those which have invaded the intra-mural tissues, whilst the manipulation necessary not infrequently disturbs injected thrombi and spreads the infection.

Progressive sterilisation as suggested by Carrel is with difficulty applied to the uterus, for it is not easy to arrange irrigating tubes so as to be sure of the antiseptic reaching every part of the infected surface, but the matter is still *sub judice*.

Conclusions.

We must, then, regard the theory of heterogenous as distinct from autogenous infection to be an exceedingly valuable one, and which once accepted will lead to a long series of investigations which may well culminate in the discovery of a specific treatment for this disease. To each city hospital where cases of puerperal fever are admitted there should be attached a keen young obstetrician in touch with a pathological laboratory and competent to undertake such surgical treatment as may appear to him advisable.

